# Learning patterns of HIV-1 resistance to broadly neutralizing antibodies with reduced subtype bias using multi-task learning

**DOI:** 10.1371/journal.pcbi.1012618

**Published:** 2024-11-20

**Authors:** Aime Bienfait Igiraneza, Panagiota Zacharopoulou, Robert Hinch, Chris Wymant, Lucie Abeler-Dörner, John Frater, Christophe Fraser

**Affiliations:** 1 Pandemic Sciences Institute, Nuffield Department of Medicine, University of Oxford, Oxford, United Kingdom; 2 Big Data Institute, Li Ka Shing Centre for Health Information and Discovery, Nuffield Department of Medicine, University of Oxford, Oxford, United Kingdom; 3 Peter Medawar Building for Pathogen Research, Nuffield Department of Medicine, University of Oxford, Oxford, United Kingdom; 4 NIHR Oxford Biomedical Research Centre, Oxford University Hospitals NHS Foundation Trust, John Radcliffe Hospital, Oxford, United Kingdom; ETH Zurich, SWITZERLAND

## Abstract

The ability to predict HIV-1 resistance to broadly neutralizing antibodies (bnAbs) will increase bnAb therapeutic benefits. Machine learning is a powerful approach for such prediction. One challenge is that some HIV-1 subtypes in currently available training datasets are underrepresented, which likely affects models’ generalizability across subtypes. A second challenge is that combinations of bnAbs are required to avoid the inevitable resistance to a single bnAb, and computationally determining optimal combinations of bnAbs is an unsolved problem. Recently, machine learning models trained using resistance outcomes for multiple antibodies at once, a strategy called multi-task learning (MTL), have been shown to improve predictions. We develop a new model and show that, beyond the boost in performance, MTL also helps address the previous two challenges. Specifically, we demonstrate empirically that MTL can mitigate bias from underrepresented subtypes, and that MTL allows the model to learn patterns of co-resistance to combinations of antibodies, thus providing tools to predict antibodies’ epitopes and to potentially select optimal bnAb combinations. Our analyses, publicly available at https://github.com/iaime/LBUM, can be adapted to other infectious diseases that are treated with antibody therapy.

## Author summary

Broadly neutralizing antibodies (bnAbs) have shown potential in treating and preventing HIV-1 infections. An important step before administering bnAbs is to determine which ones are likely to work against the HIV-1 strains that individuals or populations have. Machine learning methods have emerged as a fast and cheap way to predict HIV-1 sensitivity to bnAbs. Some of the recent methods use a technique called multi-task learning (MTL), in which data from non-broadly neutralizing antibodies are used to train models intended to predict bnAb resistance. Such models can achieve high performance, and we report that the same techniques also offer other advantages that traditional methods cannot. In particular, we first show that MTL alleviates issues arising from HIV-1 subtype imbalances in the training datasets. Then we demonstrate that MTL models provide insights into bnAb epitopes. We end with a hypothesis about how these models could be used to select optimal combinations of bnAbs. Our analyses are relevant not just for HIV-1, but also for other infectious diseases for which monoclonal antibodies are being explored as treatment.

## Introduction

Broadly neutralizing antibodies (bnAbs) exhibiting exceptional breadth and potency have revived the hope for their use in immunotherapy to prevent and to treat HIV-1 [1]. To neutralize most viruses and to prevent viral escape, bnAbs will likely be given in combinations. For example, in two separate phase 1b clinical trials, the combination of 3BNC117 and 10–1074 achieved viral suppression for roughly 20 weeks without antiretroviral therapy in 9 out of 11 individuals (pre-selected for antibody sensitivity), and in 13 out of 17 individuals (not pre-selected for antibody sensitivity), respectively [2,3]. Nonetheless, the general question of which bnAbs to administer together to achieve maximum efficacy is still outstanding.

Given that bnAbs target HIV’s envelope glycoprotein (Env), neutralization assays are traditionally used to determine the breadth and potency of different bnAbs against panels of Env-pseudotyped viruses [4]. For each pseudovirus, these experiments determine the bnAb concentration needed to reduce infectivity by 50% or 80% (i.e., IC50 or IC80, respectively). These assays are expensive and slow. In particular, when the goal is to identify bnAbs that are likely to neutralize most viruses in a given population, there is the need for scalable computational methods to predict Env sequences’ sensitivity to bnAbs.

Several machine learning (ML) models [5–12] that map Env sequences to bnAb susceptibility have been developed using neutralization data compiled in CATNAP [13]. The generalizability of these methods beyond the training data is unclear, as the training datasets have HIV-1 subtype compositions that are unrepresentative of large epidemics in sub-Saharan Africa (S1 Fig) [14], where the two thirds of people living with HIV-1 worldwide reside [15]. This is particularly worrying since susceptibility to bnAbs can be subtype-dependent [16].

Some of the most recent ML models in predicting HIV-1 resistance to many bnAbs use multi-task learning (MTL) [9,12]. The premise of MTL is that information from different but related tasks is beneficial to specific tasks of interest [17]. In this context one model is trained using neutralization outcomes for multiple antibodies at once, as opposed to only considering one antibody per model. Here we show that, in addition to a boost in performance in some cases, MTL provides solutions, at least partially, to the challenges related to data imbalances and to the selection of optimal bnAb combinations. Specifically, we empirically show that: a) MTL can mitigate bias against underrepresented HIV-1 subtypes; b) MTL allows learning patterns of co-resistance between antibodies, thus providing tools to predict antibodies’ epitopes and to potentially select optimal bnAb combinations.

## Results

### Model rationale

A common modeling choice is to align Env sequences and treat a site in the alignment as a categorical variable [5–8,10,11]. However, Env is highly variable, thus making multiple sequence alignment very challenging. Natural language processing (NLP) techniques offer alignment-free methods, which leverage the distributional hypothesis originating from linguistics [18]. The hypothesis stipulates that similar words tend to occur in similar contexts. This allows language models trained on large corpora to learn semantically meaningful vector representations of words, called word embeddings. In the case of modeling protein sequences, each amino acid can be treated as a word whose embedding is learned based on its co-occurrences with other amino acids in many sequences. Importantly, the embeddings need not be fixed for each amino acid, but can rather vary depending on the rest of the sequence, resulting in contextualized embeddings.

Following Hie *et al*’s method [19], we trained a base Env language model to learn contextualized embeddings. This task consisted in predicting each amino acid in the sequence given the rest of the sequence. The average of these embeddings across all amino acids in a sequence can be understood as the overall vector representing the sequence. Such vectors can be used to explain variations between sequences [19]. This phase of training the base model is what we call “pretraining,” which only requires Env sequences, without any neutralization data attached to them. In this work, we pretrained using 71390 Env sequences from the Los Alamos National Laboratory HIV Sequence Database (https://www.hiv.lanl.gov/). As many sequences without neutralization data are available, we hypothesized that pretraining would potentially improve the model’s generalizability, in addition to making the model learn alignment-free sequence encodings.

The second component of an input to a MTL model is the antibody of interest. Inspired by works in NLP [20–22], we represent each antibody by a unique vector. We call this vector an antibody context. Based on the distributional hypothesis, we reasoned that differences between learned antibody contexts would encode correlations between antibodies’ resistance profiles, thus offering insights into potential optimal bnAb combinations. For simplicity, we did not consider antibody sequences themselves, unlike in [12]. Instead, antibody contexts were randomly initialized and tuned using neutralization data linking antibodies to Env sequences in the training data. The resulting MTL model is what we refer to as a language-based universal model (LBUM). Further details of the model are given in *Methods*.

### No single model dominates across all bnAbs

We considered 33 bnAbs grouped in five classes depending on their epitopes: the membrane-proximal external region (MPER), the CD4 binding sites (CD4bs), the third constant region and the third variable loop (C3/V3), the first and second variable loops (V1/V2), and the “other” category for bnAbs whose epitopes do not fit in the four other classes [16,23–27]. The first two columns of Tables 1 and 2 show the class for each of the 33 bnAbs.

**Table 1:** Models’ mean AUC on IC50 data. Shown is the mean area under the receiver operating characteristic curve (AUC). Numbers between parentheses are standard deviations. The phenotype was defined using IC50 with 50 μg/mL threshold. GBM is Gradient Boosting Machines; RF is Random Forests; LBUM is language-based universal model; ENS is the ensemble model that averages predictions from GBM, RF and LBUM. The red shade means that LBUM had a better score than both RF and GBM models. The blue shade means the ensemble model scored better than all three individual models.

Class	BnAb	GBM	RF	LBUM	ENS
CD4bs	VRC07	0.81 (0.03)	0.84 (0.02)	0.95 (0.04)	0.95 (0.05)
	VRC01	0.87 (0.04)	0.87 (0.02)	0.91 (0.01)	0.92 (0.01)
	NIH45-46	0.86 (0.04)	0.83 (0.07)	0.94 (0.03)	0.93 (0.03)
	VRC-CH31	0.71 (0.09)	0.75 (0.08)	0.84 (0.07)	0.84 (0.07)
	VRC-PG04	0.78 (0.04)	0.74 (0.07)	0.85 (0.03)	0.85 (0.04)
	HJ16	0.53 (0.04)	0.56 (0.02)	0.65 (0.05)	0.59 (0.04)
	3BNC117	0.89 (0.03)	0.89 (0.04)	0.93 (0.04)	0.92 (0.03)
	VRC03	0.83 (0.05)	0.86 (0.05)	0.84 (0.05)	0.89 (0.04)
	VRC13	0.86 (0.06)	0.84 (0.07)	0.75 (0.06)	0.86 (0.07)
	b12	0.82 (0.03)	0.82 (0.04)	0.76 (0.03)	0.83 (0.04)
C3/V3	DH270.1	0.91 (0.06)	0.92 (0.04)	0.95 (0.02)	0.97 (0.03)
	VRC29.03	0.81 (0.09)	0.86 (0.08)	0.84 (0.06)	0.88 (0.05)
	DH270.5	0.92 (0.06)	0.93 (0.07)	0.95 (0.04)	0.97 (0.04)
	DH270.6	0.95 (0.03)	0.96 (0.03)	0.95 (0.03)	0.99 (0.01)
	PGT135	0.79 (0.08)	0.85 (0.04)	0.79 (0.03)	0.83 (0.05)
	PGT128	0.86 (0.05)	0.87 (0.05)	0.87 (0.04)	0.90 (0.04)
	2G12	0.92 (0.03)	0.92 (0.04)	0.85 (0.03)	0.92 (0.03)
	PGT121	0.91 (0.02)	0.91 (0.01)	0.88 (0.03)	0.93 (0.02)
	10–1074	0.97 (0.02)	0.96 (0.02)	0.89 (0.05)	0.98 (0.01)
MPER	4E10	0.68 (0.06)	0.71 (0.08)	0.81 (0.04)	0.79 (0.05)
	2F5	0.95 (0.02)	0.95 (0.01)	0.91 (0.01)	0.96 (0.01)
V1/V2	VRC26.08	0.86 (0.04)	0.86 (0.04)	0.94 (0.03)	0.94 (0.03)
	VRC26.25	0.83 (0.06)	0.85 (0.06)	0.90 (0.01)	0.90 (0.03)
	PG16	0.79 (0.05)	0.81 (0.03)	0.87 (0.02)	0.86 (0.03)
	PG9	0.82 (0.04)	0.82 (0.04)	0.87 (0.06)	0.87 (0.05)
	CH01	0.75 (0.08)	0.80 (0.07)	0.80 (0.06)	0.82 (0.06)
	PGDM1400	0.88 (0.04)	0.89 (0.03)	0.93 (0.01)	0.94 (0.02)
	VRC38.01	0.81 (0.07)	0.84 (0.06)	0.67 (0.11)	0.83 (0.08)
	PGT145	0.81 (0.04)	0.83 (0.02)	0.83 (0.05)	0.87 (0.03)
other	35O22	0.55 (0.08)	0.60 (0.02)	0.61 (0.08)	0.61 (0.02)
	VRC34.01	0.84 (0.05)	0.83 (0.06)	0.72 (0.06)	0.83 (0.05)
	PGT151	0.77 (0.04)	0.80 (0.03)	0.72 (0.08)	0.80 (0.03)
	8ANC195	0.87 (0.03)	0.88 (0.03)	0.67 (0.03)	0.87 (0.03)

**Table 2:** Models’ mean AUC on IC80 data. Shown is the mean area under the receiver operating characteristic curve (AUC). Numbers between parentheses are standard deviations. The phenotype was defined using IC80 with 1 μg/mL threshold. GBM is Gradient Boosting Machines; RF is Random Forests; LBUM is language-based universal model; ENS is the ensemble model that averages predictions from GBM, RF and LBUM. The red shade means that LBUM had a better score than both RF and GBM models. The blue shade means the ensemble model scored better than all three individual models.

Class	BnAb	GBM	RF	LBUM	ENS
CD4bs	VRC07	0.64 (0.05)	0.68 (0.04)	0.89 (0.03)	0.83 (0.05)
	VRC01	0.73 (0.04)	0.74 (0.03)	0.87 (0.03)	0.85 (0.02)
	NIH45-46	0.64 (0.06)	0.70 (0.04)	0.83 (0.05)	0.82 (0.03)
	VRC-CH31	0.59 (0.06)	0.62 (0.07)	0.83 (0.08)	0.82 (0.06)
	VRC-PG04	0.54 (0.12)	0.65 (0.05)	0.84 (0.07)	0.81 (0.09)
	HJ16	0.35 (0.13)	0.41 (0.12)	0.42 (0.07)	0.39 (0.11)
	3BNC117	0.82 (0.04)	0.78 (0.03)	0.85 (0.02)	0.84 (0.02)
	VRC03	0.75 (0.08)	0.76 (0.08)	0.85 (0.05)	0.84 (0.05)
	VRC13	0.77 (0.05)	0.73 (0.05)	0.76 (0.04)	0.81 (0.05)
	b12	0.86 (0.09)	0.91 (0.05)	0.77 (0.21)	0.90 (0.10)
C3/V3	VRC29.03	0.63 (0.21)	0.63 (0.22)	0.73 (0.19)	0.65 (0.23)
	PGT135	0.64 (0.05)	0.65 (0.12)	0.78 (0.09)	0.74 (0.08)
	PGT128	0.75 (0.04)	0.78 (0.03)	0.70 (0.03)	0.80 (0.04)
	2G12	0.53 (0.19)	0.59 (0.25)	0.79 (0.09)	0.63 (0.21)
	PGT121	0.91 (0.01)	0.91 (0.01)	0.78 (0.03)	0.93 (0.00)
	10–1074	0.91 (0.03)	0.94 (0.01)	0.79 (0.07)	0.96 (0.00)
MPER	4E10	0.55 (0.18)	0.58 (0.20)	0.91 (0.10)	0.84 (0.14)
	2F5	0.65 (0.22)	0.68 (0.18)	0.93 (0.06)	0.93 (0.04)
V1/V2	VRC26.08	0.88 (0.03)	0.86 (0.03)	0.84 (0.06)	0.90 (0.03)
	VRC26.25	0.84 (0.04)	0.84 (0.03)	0.83 (0.04)	0.87 (0.02)
	PG16	0.71 (0.08)	0.75 (0.07)	0.73 (0.05)	0.77 (0.06)
	PG9	0.74 (0.03)	0.74 (0.04)	0.74 (0.02)	0.79 (0.02)
	CH01	0.55 (0.16)	0.57 (0.09)	0.72 (0.16)	0.63 (0.17)
	PGDM1400	0.88 (0.02)	0.88 (0.02)	0.88 (0.02)	0.90 (0.01)
	VRC38.01	0.80 (0.03)	0.76 (0.05)	0.71 (0.09)	0.78 (0.06)
	PGT145	0.62 (0.02)	0.61 (0.07)	0.66 (0.03)	0.68 (0.03)
other	35O22	0.53 (0.11)	0.57 (0.16)	0.50 (0.12)	0.49 (0.07)
	VRC34.01	0.80 (0.02)	0.86 (0.05)	0.71 (0.07)	0.84 (0.02)
	PGT151	0.70 (0.07)	0.74 (0.06)	0.68 (0.13)	0.72 (0.09)
	8ANC195	0.75 (0.13)	0.79 (0.08)	0.64 (0.14)	0.75 (0.08)

Our aim was not to simply develop models that predict HIV-1 resistance to the 33 bnAbs; however, we still compared the LBUM to models developed with classical machine learning algorithms, namely random forests (RF) and gradient boosting machines (GBM), which were also used in previous publications [5–7]. We caution against comparisons to previously published performances since CATNAP data has changed over time, and preprocessing and model-selection techniques vary across publications [28].

We assessed models using three metrics: the area under the receiver operating characteristic curve (AUC), interpreted as the probability that a model ranks resistant sequences above sensitive ones; the area under the precision-recall curve (PR AUC), which measures how the model trades off precision for sensitivity, an important metric especially when resistant sequences are rare; and the binary cross-entropy (Log Loss), which measures the difference between predicted resistance probabilities and the ground truth.

We developed two sets of models based on whether IC50 or IC80 was used to define the phenotype. Since IC80 is less reported in CATNAP than IC50, we could consider only 30 bnAbs for IC80-based models instead of 33, after all the preprocessing was done (see *Methods*). The IC50-based LBUM was fine-tuned using 362 antibodies in addition to the 33 bnAbs of interest, whereas the IC80-based LBUM was fine-tuned using 99 antibodies in addition to the 30 bnAbs of interest.

On the IC50 dataset, the LBUM achieved higher AUC than both RF and GBM models did on 16 bnAbs out of 33 bnAbs (Table 1). The LBUM achieved higher PR AUC than the two other models did on 15 bnAbs out of 33 bnAbs (File S1). In terms of Log Loss, the LBUM scored better than both RF and GBM models did on 15 bnAbs out of 33 bnAbs (File S1).

On the IC80 dataset, the LBUM had a higher AUC than both RF and GBM models did on 15 bnAbs out of 30 bnAbs (Table 2). The LBUM also scored better in terms of PR AUC than both RF and GBM models did on 16 bnAbs out of 30 bnAbs (S2 File). The LBUM achieved the best Log Loss scores on 15 bnAbs out of 30 bnAbs (S2 File).

The LBUM consistently had the best performance on CD4bs and MPER bnAbs and the worst performance on “other” bnAbs compared to RF and GBM models (S2 Fig). This suggests that some epitopes may be easier to predict for the LBUM than for GBM and RF models, and vice-versa. Even within each bnAb class, there is diversity of bnAb resistance patterns [16]. This could explain why the LBUM underperformed on VRC13, b12, 10–1074, PGT121, 2G12, 8ANC195, PGT151, VRC34.01, and VRC38.01 (Tables 1 and 2). We speculate that multi-task learning did not benefit those bnAbs whose resistance profiles may be too different from those of the other bnAbs included in the training process, so much so the two bnAb-specific models, namely GBM and RF, were at an advantage. Finally, we note that all three models underperformed on HJ16 and 35O22, which was the case even in previous studies [5,12]. In addition to these bnAbs’ training data potentially being of poor quality, we conjecture that resistance to these two bnAbs is also intrinsically hard to predict.

Overall, there was no single model that consistently outperformed all other models across all bnAbs (S2 Fig). Nevertheless, averaging predicted resistance probabilities from the three models—defining the ensemble model, ENS—mitigated underperformances from individual models. Indeed, in some cases, the ensemble model could achieve higher performance than all individual models on several bnAbs (Tables 1 and 2 and S1 and S2 Files).

GBM and RF models offer some level of interpretability through variable importance, which measures how different features (alignment sites in our use-case) relatively contribute to the models’ predictions [29,30]. We found that for all bnAbs, fewer than 700 sites out of 1022 were deemed important according to RF models, while fewer than 300 sites out of 1022 were important for GBM models (S3A and S4A Figs). We defined important sites as sites given more than 0% variable importance. Hence, providing full Env to GBM and RF models was not necessary at test time as long as all important sites were provided (S3C and S4C Figs). However, the LBUM’s performance degraded when given only the same important sites as for RF and GBM models, and the longer the input sequences, the better the LBUM performed on partial Env (S3B and S4B Figs).

### Multi-task learning can mitigate HIV-1 subtype bias

Publicly available training datasets are very imbalanced in terms of HIV-1 subtypes (S1 Fig), which can compromise models’ generalizability to underrepresented subtypes, a problem we call ‘subtype bias’ hereafter. The LBUM uses, in addition to the usual bnAb data, large numbers of Env sequences with no neutralization data, and also data from antibodies not deemed as bnAbs. We hypothesized that both of these data sources help to mitigate subtype bias, because they have more balanced availability across subtypes than bnAb data. To test the two aspects separately, one would ideally vary the composition of subtypes at different training stages. However, only the IC50 datasets with subtype B and subtype C had sufficient data to run meaningful tests (S1 Fig).

**Fig 1:**
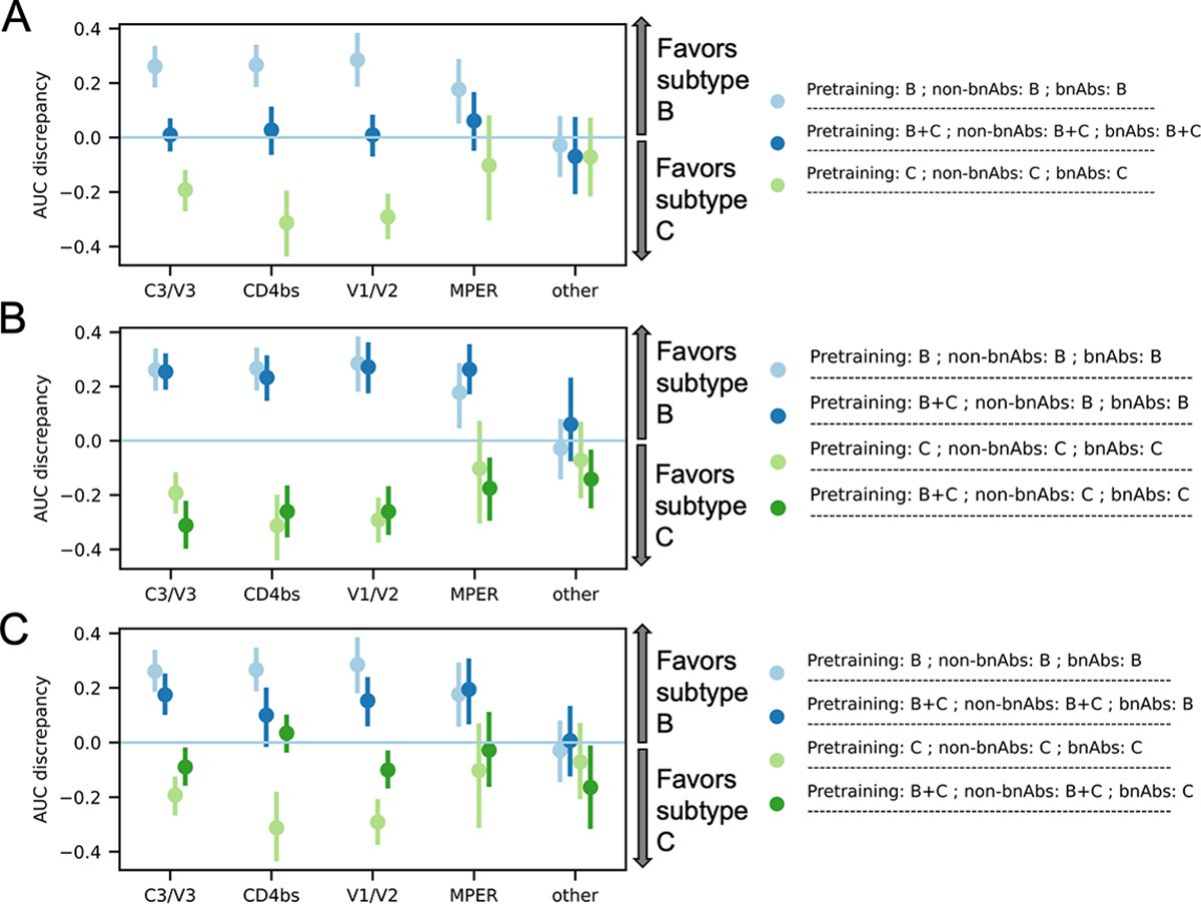
Effect of subtype representativeness on AUC. We named models according to the subtype combinations contained in the pretraining data (shown as “Pretraining”), in data on non-bnAbs (shown as “non-bnAbs”), and in bnAb data (shown as “bnAbs”). AUC discrepancy means AUC on subtype B minus AUC on subtype C. (A) shows the bias introduced by only training on one subtype, and how that bias is eliminated by more subtype diversity. (B) shows that subtype representativeness in the pretraining data reduces subtype bias only to a small extent, if at all. (C) shows how subtype representativeness in non-bnAb data reduces subtype bias. Error bars represent the 95% confidence intervals computed using 1000 bootstrap samples. Models were trained on sequences paired with phenotypes defined using IC50.

To quantify the level of subtype bias we trained two models, one with only subtype B data and one with only subtype C data. We then evaluated the models on the subtype B and subtype C bnAb testing sets separately. With the exception of “other” bnAbs (n = 4) and MPER bnAbs (n = 2), the AUC was greater by roughly 0.3 on the matched subtype than on the unmatched (Fig 1A). The model trained on both subtypes did equally well at classifying both subtypes (Fig 1A). While experimental data from antibody neutralization assays was not available for all subtypes, there was sequence data for all subtypes. To test whether using the additional sequence data improved the generalizability of the models, we re-trained the subtype-specific model but included both subtypes in the initial pretraining step. Unfortunately, using the additional sequences improved the generalizability only minimally, if at all (Fig 1B).

While neutralization data for bnAbs is limited, there is often data for other antibodies, which we label as “non-bnAbs”. We tested whether including this non-bnAb data in the training of the subtype-specific models improved their generalizability. Except on “other” and MPER bnAbs, these models showed much greater generalizability, with the difference in AUC between the two subtypes dropping to roughly 0.1 or less in many cases (Fig 1C). The “other” bnAbs’ exception could be due to the fact that the LBUM does not perform well on those four bnAbs in general (Tables 1 and 2 and S2 Fig). For the MPER bnAbs, incorporating subtype B non-bnAb data was not as impactful as expected, while we observed positive trends for subtype C (Fig 1C). This exception probably has to do with the relative quality of subtype B data relevant to these two bnAbs.

PR AUC and Log Loss generally showed similar patterns of subtype bias to those seen for AUC (S5 and S6 Figs). That is, subtype representativeness in non-bnAb data improved PR AUC on the unmatched subtype (S5C and S6C Figs), while pretraining with the subtype of interest had very minimal effects on subtype bias (S5B and S6B Figs). The exceptions for “other” and MPER bnAbs remained. We also note that Log Loss discrepancy did not change as much on C3/V3 bnAbs for subtype B, despite subtype representativeness in non-bnAb data (S6C Fig).

### Do the learned antibody contexts encode co-resistance patterns?

**Fig 2:**
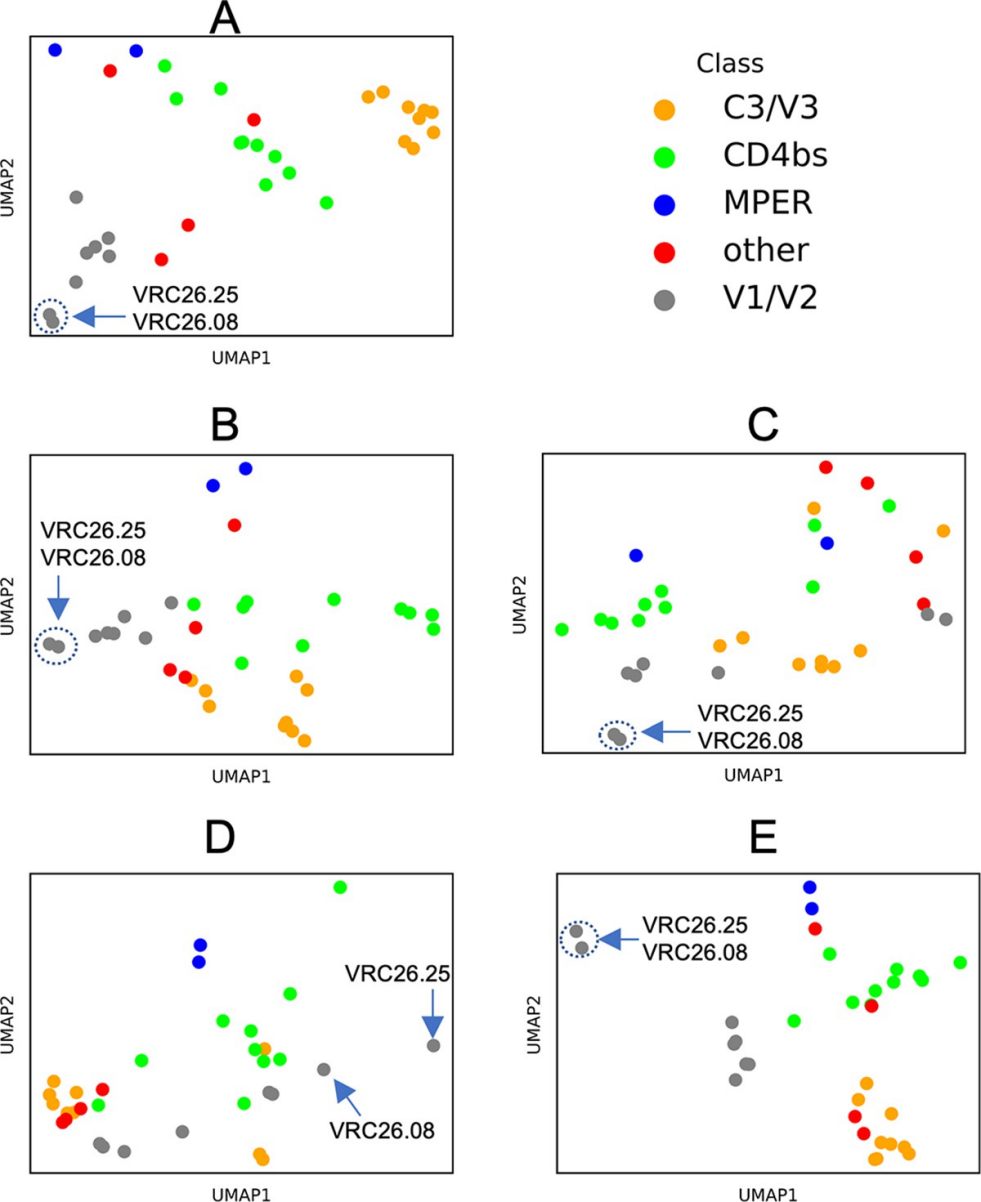
Learned antibody contexts. Antibody contexts (i.e., vector representations of antibodies) learned as part of the attention mechanism. As a result of performing 5-fold cross-validation, with 5 random seeds for each fold, 25 LBUMs were available. For each fold, we averaged antibody contexts such that 5 such contexts are remaining per bnAb. Each of the 5 subfigures represents a fold after averaging across seeds. BnAbs are color-coded according to their classes. Arrows point to some bnAbs of interest; dashed-line circles show where one arrow points to two very close bnAbs. This data is from IC50-based models.

If learned antibody contexts encode co-resistance patterns, we would expect many bnAbs targeting similar epitopes to have similar contexts, given that bnAbs targeting similar epitopes tend to have similar resistance patterns [16]. Clustering by bnAb class could be observed after projecting the dimensionality of the antibody contexts to a two-dimensional space (Fig 2A–2E). Without any further training we could predict bnAb classes solely based on the class of the closest bnAb in that context space with at least 70% accuracy (Table 3). We defined closeness between bnAbs in terms of cosine similarity, L1 distance and L2 distance between their context vectors. In at least 91% of cases, at least one of the 5 closest bnAbs belonged to the same class as the bnAb in question (Table 3), further suggesting that antibody contexts captured epitope-specific resistance patterns.

**Table 3:** Proportion of bnAbs that belonged to the same class as at least one of the closest bnAbs. The numbers between parentheses are standard deviations, since there are 25 LBUMs that resulted from performing 5-fold cross-validation, each fold having 5 models seeded differently. This data is from IC50-based models.

Number of closest bnAbs considered	Cosine similarity	L1 distance	L2 distance
1	0.71 (0.06)	0.70 (0.08)	0.70 (0.06)
2	0.80 (0.06)	0.79 (0.07)	0.81 (0.06)
3	0.86 (0.05)	0.85 (0.05)	0.85 (0.04)
4	0.89 (0.05)	0.88 (0.05)	0.89 (0.03)
5	0.92 (0.05)	0.91 (0.04)	0.91 (0.03)

Although bnAbs targeting similar epitopes generally tend to have similar resistance profiles, that is not always the case. Indeed, we observed outliers in class clusters (Fig 2). Such within-class dissimilarities could imply different resistance patterns among bnAbs targeting similar epitopes. A known example of dissimilar patterns within the V1/V2 class was captured by learned bnAb contexts: VRC26.08 and VRC26.25 clustered away from the rest of V1/V2 bnAbs (Fig 2A, 2B, 2C and 2E). Contrary to the rest of V1/V2 bnAbs, the potency of CAP256-VRC26 bnAbs, which include the two bnAbs, is known to be inversely dependent on the presence of a glycan at the N160 position in Env [31]. Finally, we note that some bnAbs appeared to have similar bnAb contexts despite targeting different epitopes (Fig 2). Whether such cases imply cross-class resistance correlation is an interesting question, which we leave for future work.

## Discussion

In summary, we developed a model to predict the neutralization of different HIV-1 Env sequences by different broadly neutralizing antibodies (bnAbs). Our model, which we named a language-based universal model (LBUM), is a type of multi-task learning (MTL) model. The LBUM was pretrained using Env sequences with no associated neutralization data and fine-tuned with Env sequences with both non-bnAb and bnAb outcome data. We first showed that the LBUM’s performance is comparable to that of Gradient Boosting Machine (GBM) models and Random Forest (RF) models, with some improvements over both methods (Tables 1 and 2 and S2 Fig and S1 and S2 Files). Unlike the other two methods, the LBUM does not require aligning input Env sequences, which is an advantage given the incredible variability of Env, including structural variability that makes alignment challenging. As for previous methods, all models in this work were trained to predict *in vitro* bnAb resistance: we did not validate them with clinical outcomes, and we relied on data showing correlations between *in vitro* susceptibility to bnAbs and *in vivo* outcomes [32].

A thorough systematic comparison between published methods requires testing different combinations of preprocessing techniques, feature selection methods and learning algorithms. In this work, we only compared learning algorithms applied on full Env sequences with neutralization data preprocessed similarly. We compared the LBUM to both RF and GBM models because both boosting trees and RF underlie recently published methods that do not use neural networks [5,7].

The most common subtypes in sub-Saharan Africa are A1, C, D and several circulating recombinant forms (CRFs) [14]. CATNAP, from which most of the training datasets come, has mostly subtype B and subtype C sequences (S1 Fig). This subtype mismatch is problematic because, as we have shown, models do not necessarily generalize across subtypes (Figs 1A, S5A and S6A). MTL and pretraining give access to more data with potentially more subtype representativeness. Although no solution trumps having all subtypes represented in bnAb data, our results suggest that MTL can alleviate subtype bias if neutralization data with all subtypes is available for antibodies not considered bnAbs (Figs 1C, S5C and S6C).

We introduced the concept of antibody contexts, which we defined as vector representations unique to each antibody and updated during the fine-tuning process. We showed that bnAbs targeting similar epitopes tended to have similar contexts, to such an extent that we could use closeness between antibody contexts to predict antibody epitopes (Table 3). In this regard, our methods can be used to generate hypotheses about epitopes targeted by new antibodies, as long as relevant neutralization data is part of the LBUM’s training data. Nonetheless, some bnAbs had distant contexts despite targeting similar epitopes (Fig 2 and Table 3), and we highlighted a known example that supports our hypothesis that differences in antibody contexts may capture differences in resistance profiles. A possible limitation is that negatively correlated resistance profiles can possibly lead to similar bnAb contexts, the same way antonyms can have similar word embeddings in the English language [33]. Nonetheless, we assumed that such cases were rare if present at all, given that most bnAb contexts tended to cluster per bnAb class (Table 3). Analyses of structural data on antibodies and their respective targets on Env will help further show the extent to which antibody contexts capture co-resistance patterns.

An interesting extension of our methods could be to pretrain using generic protein language models, such as those in the BERT and ESM families [34,35]. We expect MTL models’ performance to increase as their size increases along with the increase in the quantity and diversity of their training data. We chose small architectures because of computational requirements imposed by deep neural network models and because of the availability of only small amounts of data on which to fine-tune.

The potential of MTL revealed in our study addresses key challenges in HIV-1 vaccine research. All models developed in this work, along with the used code, can be found at https://github.com/iaime/LBUM. The framework presented here is a starting point towards designing effective immunotherapies. We hope that our analyses can be relevant to other infectious diseases for which monoclonal antibodies are being explored as therapeutic solutions.

## Materials and methods

### Data preprocessing

We binarized the neutralization outcome—resistant or sensitive—i.e. we aimed to predict whether positive neutralization is observed within a certain range of antibody concentrations. This is because the main use-case envisioned for our models is the identification of bnAbs that are likely to neutralize most viruses in given populations. Once bnAbs with largest coverage are identified, other methods will need to be used to determine the bnAbs’ exact potencies.

We determined the phenotype based on IC50 or IC80. We transformed left-censored IC values to the detection threshold. That is, <x values became x. Since CATNAP Env sequences could have multiple IC50 or IC80 values from different studies, we calculated the geometric mean whenever more than one value was available, as long as none of the values was right-censored. If any reported IC value for a sequence-antibody pair was right-censored, the sequence was deemed resistant to the antibody in terms of IC50 or IC80, accordingly. For models trained with IC50 data, sensitive sequences had no right-censored IC50 values and the geometric mean IC50 was less than 50 μg/mL. This cutoff was selected because it is the most used in the literature and it is the most common detection threshold in CATNAP. For models trained with IC80 data, sensitive sequences had no right-censored IC80 values and the geometric mean IC80 was less than 1 μg/mL, a cutoff inspired by the AMP trials done on VRC01 [32].

All models were trained using only sequences that are 800 to 900 amino acid long (ignoring non-amino-acid characters) to match the expected length of a full Env sequence. Nonetheless, our models still accept partial Env although performance can degrade (S3B, S3C, S4B and S4C Figs). Part of our analysis compared our model to random forests (RF) and gradient boosting machines (GBM) models. Since RF and GBM models required aligned sequences, we used the alignment provided in CATNAP to one-hot encode sequences. That is, each amino acid was represented as a vector of all zeros except a 1 at the index of that amino acid. For non-amino acid characters, the vector was all zeros. The LBUM did not require aligning sequences, and all non-amino acid characters were removed from their input sequences.

### Language-based universal model (LBUM)

The overall architecture of the proposed LBUM is rationalized in the *Results* section. There were two main steps in the development of the LBUM, namely pretraining and fine-tuning.

### Pretraining

First, we pretrained the model as a two-layer bidirectional Long Short-Term Memory (LSTM) model [36]. More specifically, the outputs of the concatenation layers for each site in the sequence were input into a dense layer with a softmax activation function, which outputs a probability distribution over the 20 amino acids and the following tokens: <start>, <end>, <unknown>, <mask>.

For a single site in a sequence, an LSTM cell has three main gates, namely the forget gate, the input gate and the output gate. We denote these by *F*, *I* and *O*, respectively. We label weight matrices *W* and bias vectors *b* in those three gates with superscripts *F*, *I* and *O*, accordingly. For each site *t*, an LSTM cell outputs two vectors: the hidden state *h*^*t*^ and the cell state *c*^*t*^, which can be viewed as short-term memory and long-term memory, respectively. The hidden states of the last forward and backward LSTM layers are concatenated in the end to produce vectors fed into subsequent layers. A cell takes three inputs: the hidden state and the cell state from the previous site *t*−1 (i.e., *h*^*t*−1^ and *c*^*t*−1^), and an input vector corresponding to the current site *t*. For the first two backward and forward layers, this input vector is the token’s embedding while for the second forward and backward LSTM layers, the input vector is the hidden state (*h*^*t*^) from the corresponding lower layer. We denote the input vector by *x*^*t*^. In summary, the following computations take place in an LSTM cell:

$$F^{t}=\sigma ({W_{h}^{F}h}^{t-1}+W_{x}^{F}x^{t}+b^{F})$$

$$I^{t}=\sigma ({W_{h}^{I}h}^{t-1}+W_{x}^{I}x^{t}+b^{I})$$

$$O^{t}=\sigma ({W_{h}^{O}h}^{t-1}+W_{x}^{O}x^{t}+b^{O})$$

$$c^{t}=F^{t}⊗c^{t-1}+I^{t}⊗tanh({W_{h}^{c}h}^{t-1}+W_{x}^{c}x^{t}+b^{c})$$

$$h^{t}=O^{t}⊗tanh(c^{t})$$

Where ⊗ means element-wise multiplication, *W*^*c*^ and *b*^*c*^ denote another set of weights and biases, *tanh* is the hyperbolic tangent function, and *σ* is the sigmoid function.

For pretraining, we maintained the same hyperparameters used by Hie *et al* [19]. Specifically, we optimized categorical cross-entropy using the Adam algorithm with the learning rate set to 0.001. The dimension of the hidden state and that of tokens’ embeddings were respectively set to 512 and 20. Dimensions of the other vectors and matrices could be derived automatically. All other hyperparameters for the pretraining phase were left to their default values in Tensorflow Keras (v2.12.0). We pretrained for 50 epochs, each epoch corresponding to the predictions for all sites in all training sequences. The model with the lowest cross-entropy loss was used in downstream tuning. For the subtype analysis where we needed to pretrain on subtype B and subtype C sequences only, we balanced the dataset by oversampling the minority subtype.

### Fine-tuning

After pretraining, we fine-tuned the LBUM using data on 362 antibodies in addition to the 33 bnAbs of interest for the IC50-based models, while we used 99 antibodies in addition to the 30 bnAbs of interest for the IC80-based models. The drop in the number of antibodies was because CATNAP has fewer IC80 values than IC50 values. Antibody context vectors were incorporated through an attention mechanism that was a combination of at least three methods [20–22]. Below we detail the attention layer.

Let *C*_*t*_ be the context of an antibody *t*. Let *E*_*j*_ be the embedding of a token *j* in a sequence *x* of length *n* (including <start>, <end>, <unknown>, <mask> tokens). The attention weight *a*_*j*_ to the token *j* given the antibody *t* context was calculated as follows:

$$R_{j}=tanh(W\;E_{j}+b)$$

$$D_{j}=R_{j}∙C_{t}$$

$$a_{j}=\frac{e^{D_{j}}}{\sum_{i=1}^{n}e^{D_{i}}}$$

where *W* and *b* are weight matrix and bias vector, respectively, and *tanh* is the hyperbolic tangent used as an activation function. We note that $\sum_{i=1}^{n}a_{i}=1$ for each sequence. The weighted average embedding $E=\sum_{i=1}^{n}a_{i}\times E_{i}$ was then input to a dense output layer. We added two dropout layers, one before the attention layer and another before the final dense layer. The dropout rate was determined via hyperparameter search described in another section. To visualize the antibody contexts in Fig 2, we used the Uniform Manifold Approximation and Projection algorithm (UMAP) [37]. As a regularization technique, we added a secondary output layer in the LBUM that directly predicts log_10_(IC50) or log_10_ (IC80), depending on whether IC50 or IC80 was used to define the phenotype, respectively. However, sequence-antibody pairs with IC50 (or IC80) beyond the detection threshold (i.e., right-censored IC50 or right-censored IC80) did not contribute towards the training of the regression branch. A question not addressed here is how to incorporate censored data into the training data of models that predict IC50 or IC80. For now, we recommend against making predictions with the regression branch of the trained model, as it cannot be relied on given its biased training data. The LBUM’s overall loss function was simply the weighted average of binary cross-entropy and mean squared error:

$$-\frac{\beta }{n}\sum_{i=1}^{n}\left(y_{i}\;log(y_{i}^{p})+({1-y}_{i})log({1-y}_{i}^{p})\right)+\frac{1-\beta }{n}\sum_{i=1}^{n}{\left(l_{i}-l_{i}^{p}\right)}^{2}$$

where *n* = 32 and is the number of sequence-phenotype pairs per training batch, *y*_*i*_ = 0 if the sequence is sensitive to the bnAb in question else *y*_*i*_ = 1, $y_{i}^{p}$ is the probability of resistance predicted by the LBUM, *l*_*i*_ is the corresponding log_10_(IC50) or log_10_ (IC80), $l_{i}^{p}$ is the predicted log_10_(IC50) or log_10_ (IC80), and *β* weights the tradeoff between the two losses.

During fine-tuning, we froze all pretrained layers except the last forward and backward LSTM layers, and we applied early stopping with a 10-epoch patience. At inference time, we averaged predictions from running 10 forward passes with dropout turned on. For each fold from 5-fold cross-validation (see cross-validation section), we fine-tuned 5 models, each with a different random seed. Thus, at inference time, for each fold, we also averaged predictions from the 5 models (i.e., we averaged along both seeds and dropout forward passes). To balance the fine-tuning data, we oversampled the minority phenotype. For the subtype analysis where we fine-tuned on subtype B and subtype C data only, we balanced the data in terms of both subtype and phenotype.

### GBM and RF models, cross-validation and hyperparameter search

Both GBM and RF build ensemble models based on decision trees. For complete mathematical descriptions of GBM and RF, we refer to [30] and [29], respectively. 5-fold nested cross-validation was used to select and evaluate both types of models. That is, data was split into 5 folds, with each fold being in turn reserved for testing only, resulting in a total of 5 models. For each split, an additional 5-fold cross-validation (hence “nested cross-validation”) was performed on the training set to select hyperparameters. For each split, we considered 10 random combinations of hyperparameters shown in Table 4. Log Loss was used to select the best classifiers. Both GBM and RF models were implemented using scikit-learn (v1.1.1) [38].

**Table 4:** Hyperparameters considered in the development of different models.

Model type	Hyperparameters
Random Forests	max depth: 1, 2, 3, 4, 5 max features: 0.03, 0.1, 0.2, 0.3, 0.5 number of trees: 10, 50, 100, 500, 1000
Gradient Boosting Machines	learning rate: 0.001, 0.01, 0.05, 0.1, 0.2 max features: 0.03, 0.1, 0.2, 0.3, 0.5 max depth: 1, 2, 3, 4, 5 number of trees: 10, 50, 100, 500, 1000
Language-based universal model	learning rate: 0.0001, 0.0003, 0.001, 0.003 antibody context dimension: 32, 64, 128, 256 dropout rate: 0.1, 0.2, 0.3, 0.4, 0.5 number of pretrained layers to unfreeze: 0, 2, 4, all classification loss weight (i.e. β): 0.5, 0.6, 0.7, 0.8, 0.9, 1

For the LBUM, we performed 5-fold cross-validation, although not nested as for GBM and RF. For each fold, we determined optimal hyperparameters on the training set using Bayesian optimization implemented in KerasTuner [39]. Specifically, we tuned the learning rate for the fine-tuning phase, the dimension of antibody context vectors, the dropout rate, the number of pretrained layers to unfreeze during fine-tuning, and the weights for the classification and regression output branches of the LBUM. Considered values for these hyperparameters are shown in Table 4. Values that achieved the lowest binary cross-entropy within 10 trials were chosen for the final model. All the other hyperparameters of the LBUM were set to default values in Tensorflow Keras (v2.12.0).

All reported predictive performance metrics were computed using 5-fold cross-validation. Thus, the shown results are out-of-sample results. Specifically, for all three models, we tested the models on one of the 5 folds that was used to perform neither hyperparameter tuning nor training. The process was repeated 5 times, considering each fold as the test set in each turn. For the subtype analysis shown in Figs 1, S5 and S6, we followed the same training and testing strategy as for the full models. Then for each bnAb and each test fold, we calculated differences in performance between subtype B test data and subtype C test data. To perform the bootstrap in those three figures, we did not refit models to each bootstrap dataset. Instead, to calculate the mean and the 95% confidence interval for each bnAb class, we used 1000 samples of the discrepancies calculated on the bnAb-level test sets.

## Supporting information

S1 Fig CATNAP data. Shown are the counts of Env sequences for which IC50 values were available for each of the 33 bnAbs we considered. Distributions per subtype are color-coded. BnAbs are also color-coded according to the class they belong to.
(TIFF)

S2 Fig Models’ performance per bnAb class. Shown are the area under the receiver operating characteristic curve (AUC) (A and B), the area under the precision-recall curve (PR_AUC) (C and D), and the binary cross entropy (LOG_LOSS) (E and F). A, C and E show performance for IC50-based models while B, D, and F show performance for IC80-based models. The dotted line in subfigures A and B corresponds to AUC of a random classifier.
(TIFF)

S3 Fig IC50-based models and important sites. (A) shows the number of important sites according to RF and GBM models. Error bars represent standard deviations, given that for each bnAb there are 5 models resulting from performing 5-fold cross-validation. We defined important sites as sites given >0% variable importance by the model in question. (B) shows the area under the receiver operating characteristic curve (AUC) of the LBUM when given full Env (LBUM full), when given only important sites according to RF (LBUM partial (RF)), and when given only important sites according to GBM (LBUM partial (GBM)). Error bars represent standard deviations. The horizontal line is the 0.5 marker, which represents the AUC of a random model. (C) shows the AUC of RF and GBM when given full Env (RF full and GBM full), the AUC of both models when given only important sites (RF partial and GBM partial), and the AUC of both models when given random sites, but as many as there are important sites (RF random and GBM random). Performance on non-full Env was calculated using the same models used for full Env. That is, models were not re-trained, but only test sequences were modified by removing unimportant sites. For GBM and RF, removing sites meant zeroing all elements of corresponding one-hot encodings, without changing the size of the input alignment. Models were trained on sequences paired with phenotypes defined using IC50.
(TIFF)

S4 Fig IC80-based models and important sites. (A) shows the number of important sites according to RF and GBM models. Error bars represent standard deviations, given that for each bnAb there are 5 models resulting from performing 5-fold cross-validation. We defined important sites as sites given >0% variable importance by the model in question. (B) shows the area under the receiver operating characteristic curve (AUC) of the LBUM when given full Env (LBUM full), when given only important sites according to RF (LBUM partial (RF)), and when given only important sites according to GBM (LBUM partial (GBM)). Error bars represent standard deviations. The horizontal line is the 0.5 marker, which represents the AUC of a random model. (C) shows the AUC of RF and GBM when given full Env (RF full and GBM full), the AUC of both models when given only important sites (RF partial and GBM partial), and the AUC of both models when given random sites, but as many as there are important sites (RF random and GBM random). Performance on non-full Env was calculated using the same models used for full Env. That is, models were not re-trained, but only test sequences were modified by removing unimportant sites. For GBM and RF, removing sites meant zeroing all elements of corresponding one-hot encodings, without changing the size of the input alignment. Models were trained on sequences paired with phenotypes defined using IC80.
(TIFF)

S5 Fig Effect of subtype representativeness on PR AUC. We named models according to subtype combinations contained in the pretraining data (shown as “Pretraining”), in data on non-bnAbs (shown as “non-bnAbs”), and in bnAb data (shown as “bnAbs”). PR AUC discrepancy means PR AUC on subtype B minus PR AUC on subtype C. (A) shows the bias introduced by only training on one subtype, and how that bias is eliminated by more subtype diversity. (B) shows that subtype representativeness in the pretraining data reduces subtype bias only to a small extent, if at all. (C) shows how subtype representativeness in non-bnAb data reduces subtype bias. Error bars represent the 95% confidence intervals computed using 1000 bootstrap samples. Models were trained on sequences paired with phenotypes defined using IC50.
(TIFF)

S6 Fig Effect of subtype representativeness on Log Loss. We named models according to subtype combinations contained in the pretraining data (shown as “Pretraining”), in data on non-bnAbs (shown as “non-bnAbs”), and in bnAb data (shown as “bnAbs”). Log Loss discrepancy means Log Loss on subtype C minus Log Loss on subtype B. (A) shows the bias introduced by only training on one subtype, and how that bias is eliminated by more subtype diversity. (B) shows that subtype representativeness in the pretraining data reduces subtype bias only to a small extent, if at all. (C) shows how subtype representativeness in non-bnAb data reduces subtype bias. Error bars represent the 95% confidence intervals computed using 1000 bootstrap samples. Models were trained on sequences paired with phenotypes defined using IC50.
(TIFF)

S1 File Models’ performance on IC50 data. The area under the precision-recall curve (PR AUC) and the binary cross-entropy (Log Loss) are reported. Models were trained on sequences paired with phenotypes defined using IC50. GBM is Gradient Boosting Machines; RF is Random Forests; LBUM is language-based universal model; ENS is the ensemble model that averages predictions from GBM, RF and LBUM. The red shade means that LBUM had a better score than both RF and GBM models. The blue shade means the ensemble model scored better than all three individual models. Numbers between parentheses are standard deviations.
(XLSX)

S2 File Models’ performance on IC80 data. The area under the precision-recall curve (PR AUC) and the binary cross-entropy (Log Loss) are reported. Models were trained on sequences paired with phenotypes defined using IC80. GBM is Gradient Boosting Machines; RF is Random Forests; LBUM is language-based universal model; ENS is the ensemble model that averages predictions from GBM, RF and LBUM. The red shade means that LBUM had a better score than both RF and GBM models. The blue shade means the ensemble model scored better than all three individual models. Numbers between parentheses are standard deviations.
(XLSX)

S3 File Important sites for IC50-based models. Reported are important sites, defined as sites with >0% variable importance. The Excel file contains 4 sheets, 2 for GBM and 2 for RF. For each model, one sheet shows sites in terms of HXB2 coordinates while the other sheet shows the same sites but as CATNAP alignment’s coordinates. For each bnAb, there were 5 models resulting from 5-fold cross-validation, hence the 5 columns per bnAb. Sites are 1-indexed (i.e., the first site is numbered 1). The number between parentheses is the variable importance for the site. Models were trained on sequences paired with phenotypes defined using IC50.
(XLSX)

S4 File Important sites for IC80-based models. Reported are important sites, defined as sites with >0% variable importance. The Excel file contains 4 sheets, 2 for GBM and 2 for RF. For each model, one sheet shows sites in terms of HXB2 coordinates while the other sheet shows the same sites but as CATNAP alignment’s coordinates. For each bnAb, there were 5 models resulting from 5-fold cross-validation, hence the 5 columns per bnAb. Sites are 1-indexed (i.e., the first site is numbered 1). The number between parentheses is the variable importance for the site. Models were trained on sequences paired with phenotypes defined using IC80.
(XLSX)
