# Supplementary figures and images for: Learning patterns of HIV-1 resistance to broadly neutralizing antibodies with reduced subtype bias using multi-task learning
Source: PLoS Comput Biol. 2024 Nov 20;20(11):e1012618. doi: 10.1371/journal.pcbi.1012618 (PMC11616810; doi:10.1371/journal.pcbi.1012618)

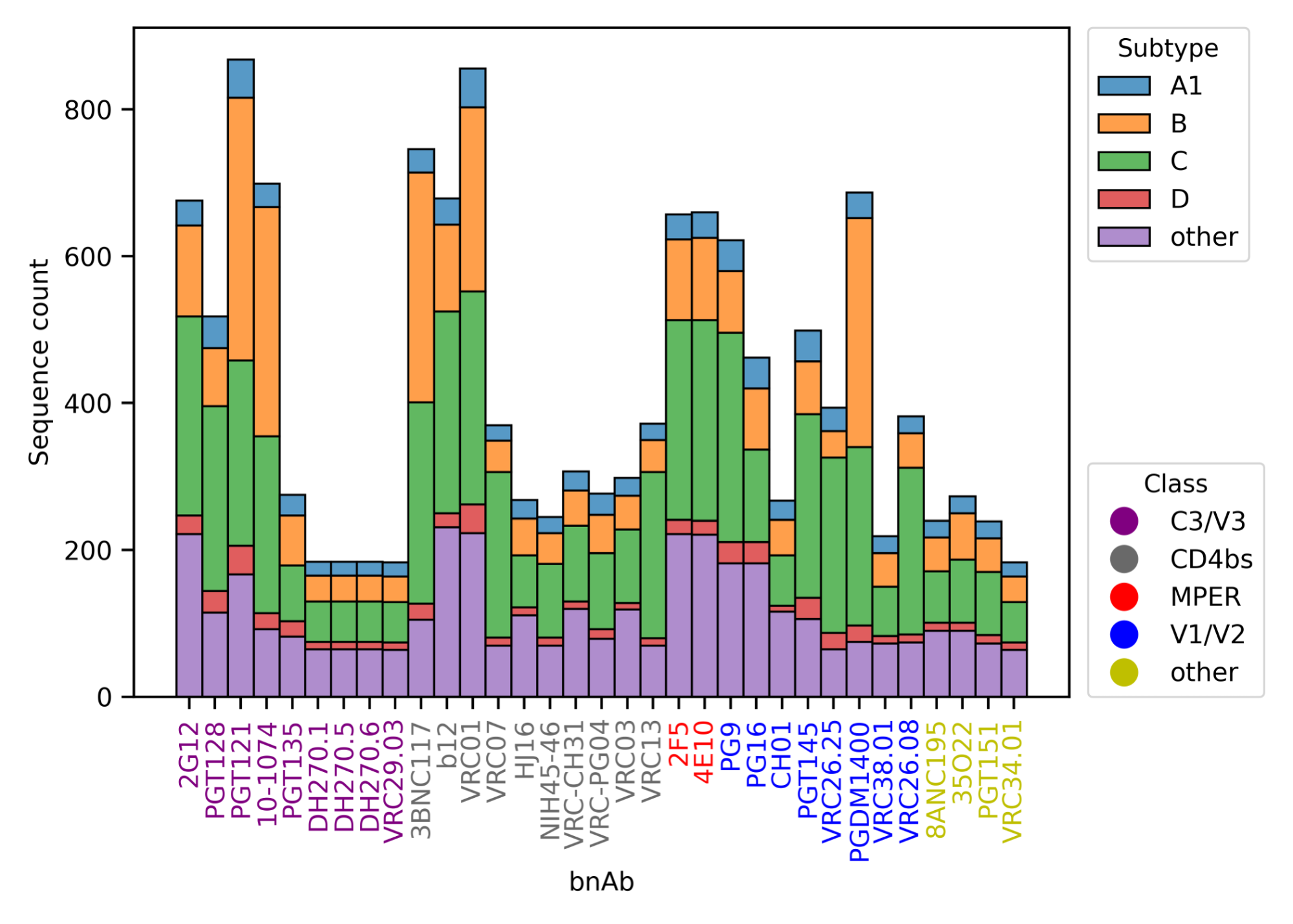

Supplement: S1 Fig — Shown are the counts of Env sequences for which IC50 values were available for each of the 33 bnAbs we considered. Distributions per subtype are color-coded. BnAbs are also color-coded according to the class they belong to. (TIFF) [file pcbi.1012618.s001.tiff]

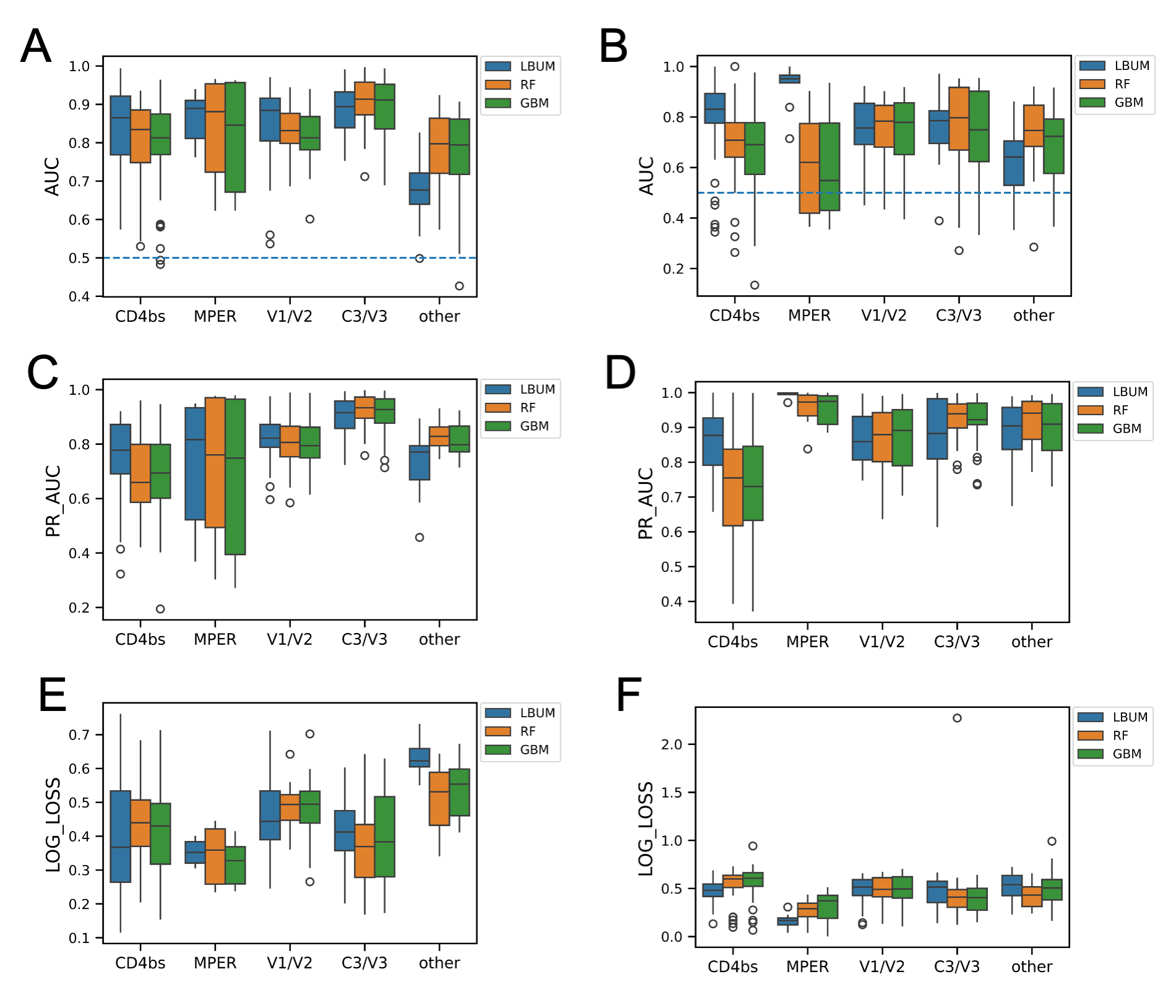

Supplement: S2 Fig — Shown are the area under the receiver operating characteristic curve (AUC) (A and B), the area under the precision-recall curve (PR_AUC) (C and D), and the binary cross entropy (LOG_LOSS) (E and F). A, C and E show performance for IC50-based models while B, D, and F show performance for IC80-based models. The dotted line in subfigures A and B corresponds to AUC of a random classifier. (TIFF) [file pcbi.1012618.s002.tiff]

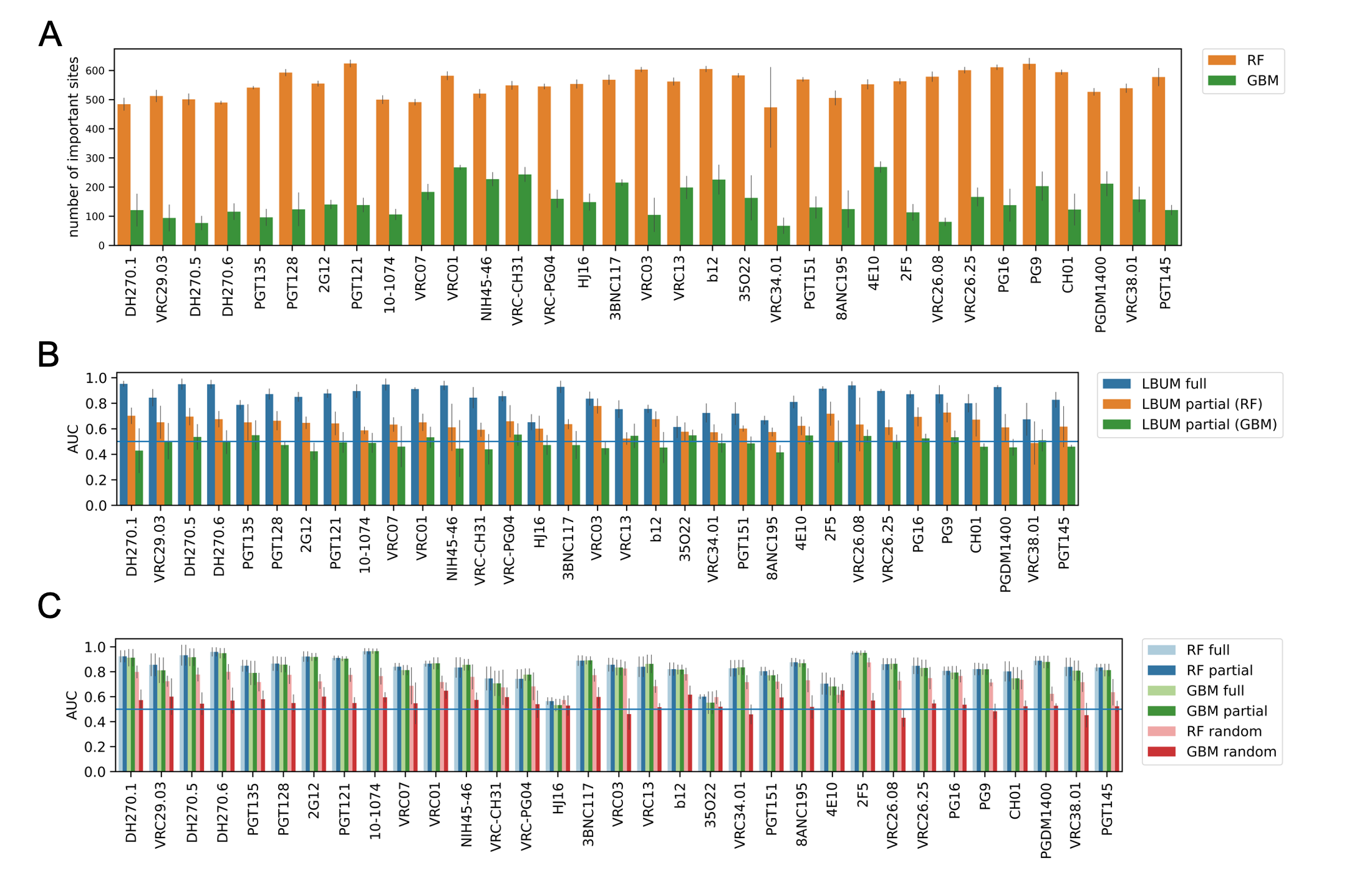

Supplement: S3 Fig — (A) shows the number of important sites according to RF and GBM models. Error bars represent standard deviations, given that for each bnAb there are 5 models resulting from performing 5-fold cross-validation. We defined important sites as sites given >0% variable importance by the model in question. (B) shows the area under the receiver operating characteristic curve (AUC) of the LBUM when given full Env (LBUM full), when given only important sites according to RF (LBUM partial (RF)), and when given only important sites according to GBM (LBUM partial (GBM)). Error bars represent standard deviations. The horizontal line is the 0.5 marker, which represents the AUC of a random model. (C) shows the AUC of RF and GBM when given full Env (RF full and GBM full), the AUC of both models when given only important sites (RF partial and GBM partial), and the AUC of both models when given random sites, but as many as there are important sites (RF random and GBM random). Performance on non-full Env was calculated using the same models used for full Env. That is, models were not re-trained, but only test sequences were modified by removing unimportant sites. For GBM and RF, removing sites meant zeroing all elements of corresponding one-hot encodings, without changing the size of the input alignment. Models were trained on sequences paired with phenotypes defined using IC50. (TIFF) [file pcbi.1012618.s003.tiff]

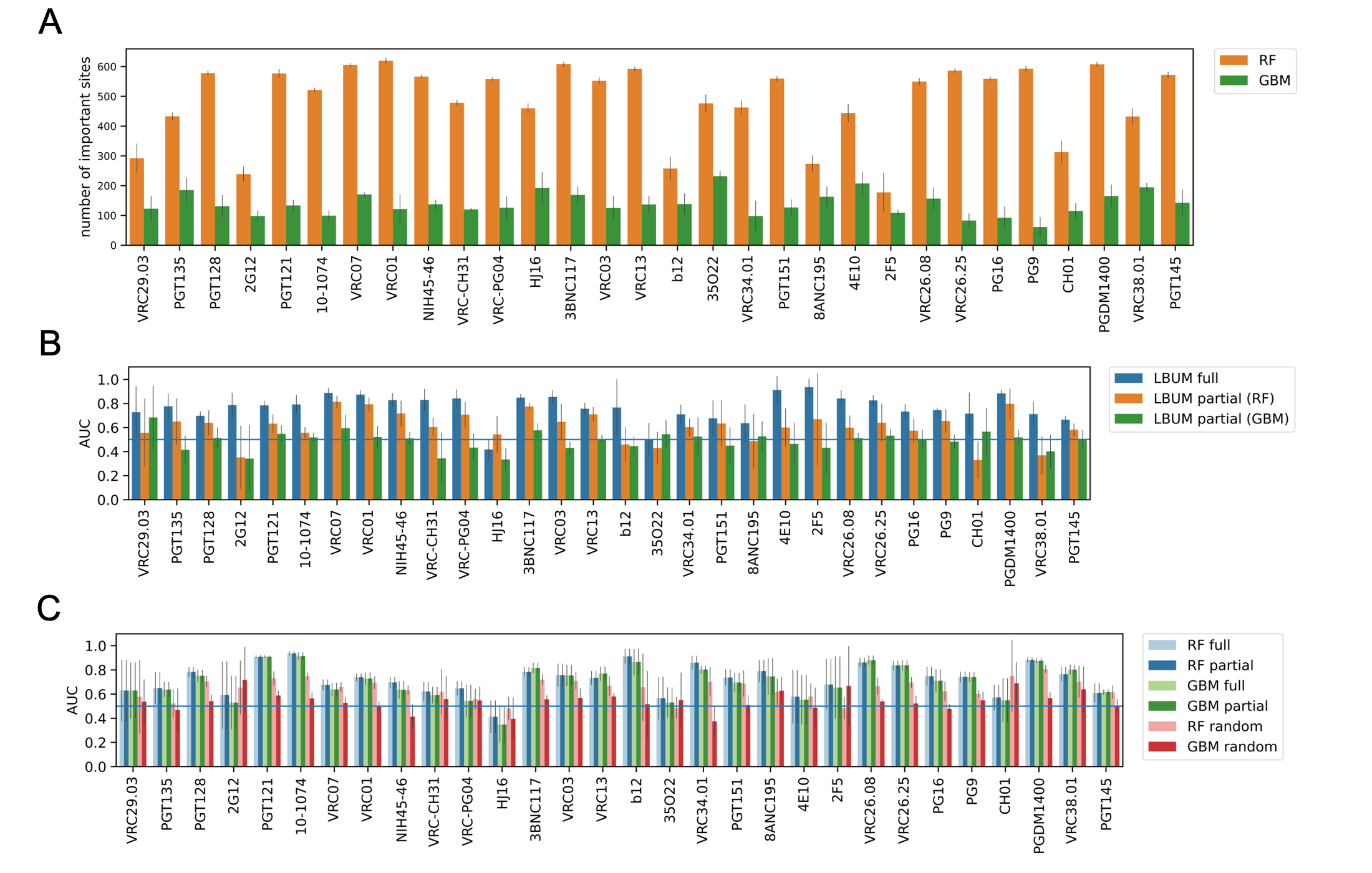

Supplement: S4 Fig — (A) shows the number of important sites according to RF and GBM models. Error bars represent standard deviations, given that for each bnAb there are 5 models resulting from performing 5-fold cross-validation. We defined important sites as sites given >0% variable importance by the model in question. (B) shows the area under the receiver operating characteristic curve (AUC) of the LBUM when given full Env (LBUM full), when given only important sites according to RF (LBUM partial (RF)), and when given only important sites according to GBM (LBUM partial (GBM)). Error bars represent standard deviations. The horizontal line is the 0.5 marker, which represents the AUC of a random model. (C) shows the AUC of RF and GBM when given full Env (RF full and GBM full), the AUC of both models when given only important sites (RF partial and GBM partial), and the AUC of both models when given random sites, but as many as there are important sites (RF random and GBM random). Performance on non-full Env was calculated using the same models used for full Env. That is, models were not re-trained, but only test sequences were modified by removing unimportant sites. For GBM and RF, removing sites meant zeroing all elements of corresponding one-hot encodings, without changing the size of the input alignment. Models were trained on sequences paired with phenotypes defined using IC80. (TIFF) [file pcbi.1012618.s004.tiff]

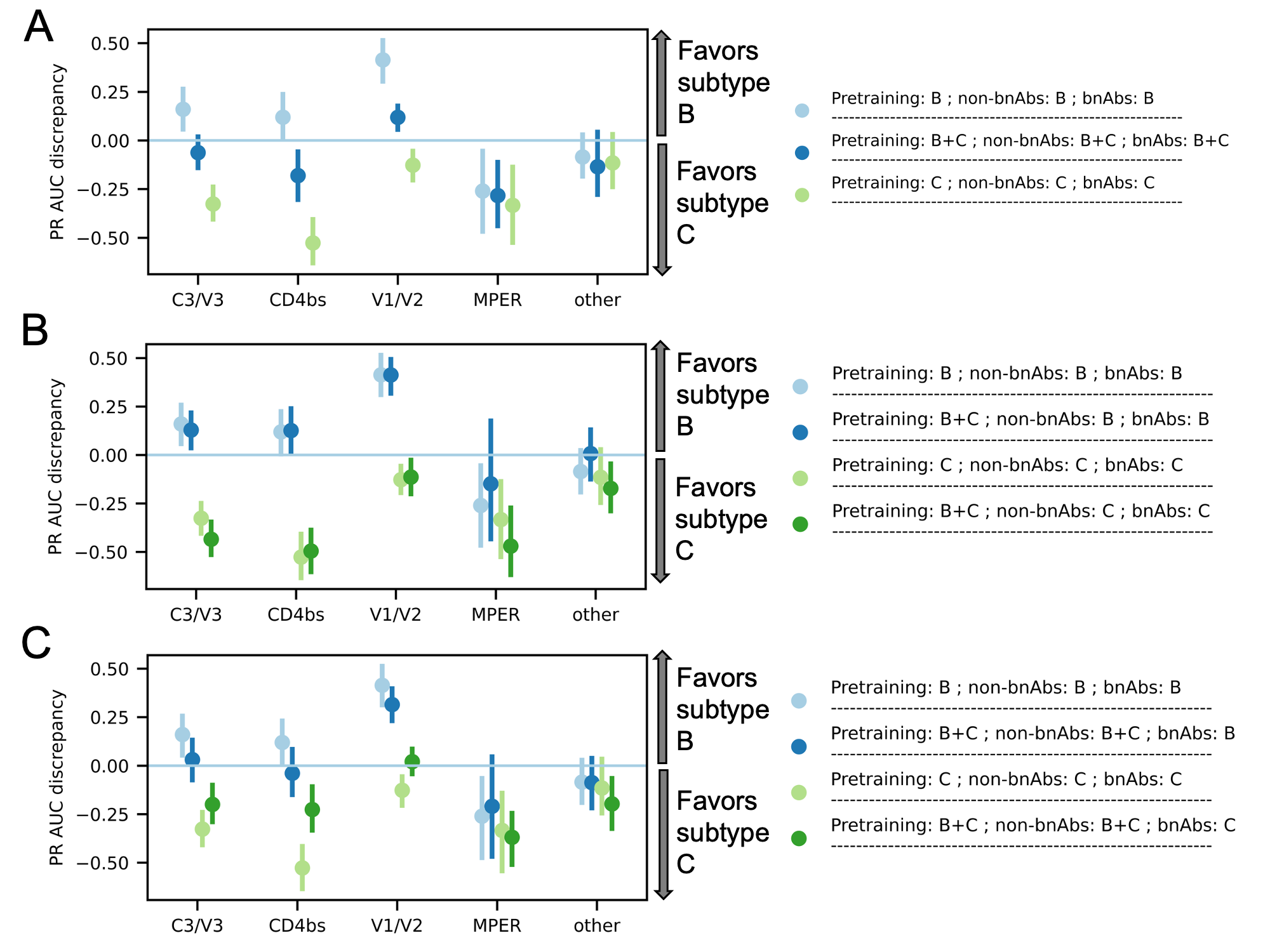

Supplement: S5 Fig — We named models according to subtype combinations contained in the pretraining data (shown as “Pretraining”), in data on non-bnAbs (shown as “non-bnAbs”), and in bnAb data (shown as “bnAbs”). PR AUC discrepancy means PR AUC on subtype B minus PR AUC on subtype C. (A) shows the bias introduced by only training on one subtype, and how that bias is eliminated by more subtype diversity. (B) shows that subtype representativeness in the pretraining data reduces subtype bias only to a small extent, if at all. (C) shows how subtype representativeness in non-bnAb data reduces subtype bias. Error bars represent the 95% confidence intervals computed using 1000 bootstrap samples. Models were trained on sequences paired with phenotypes defined using IC50. (TIFF) [file pcbi.1012618.s005.tiff]

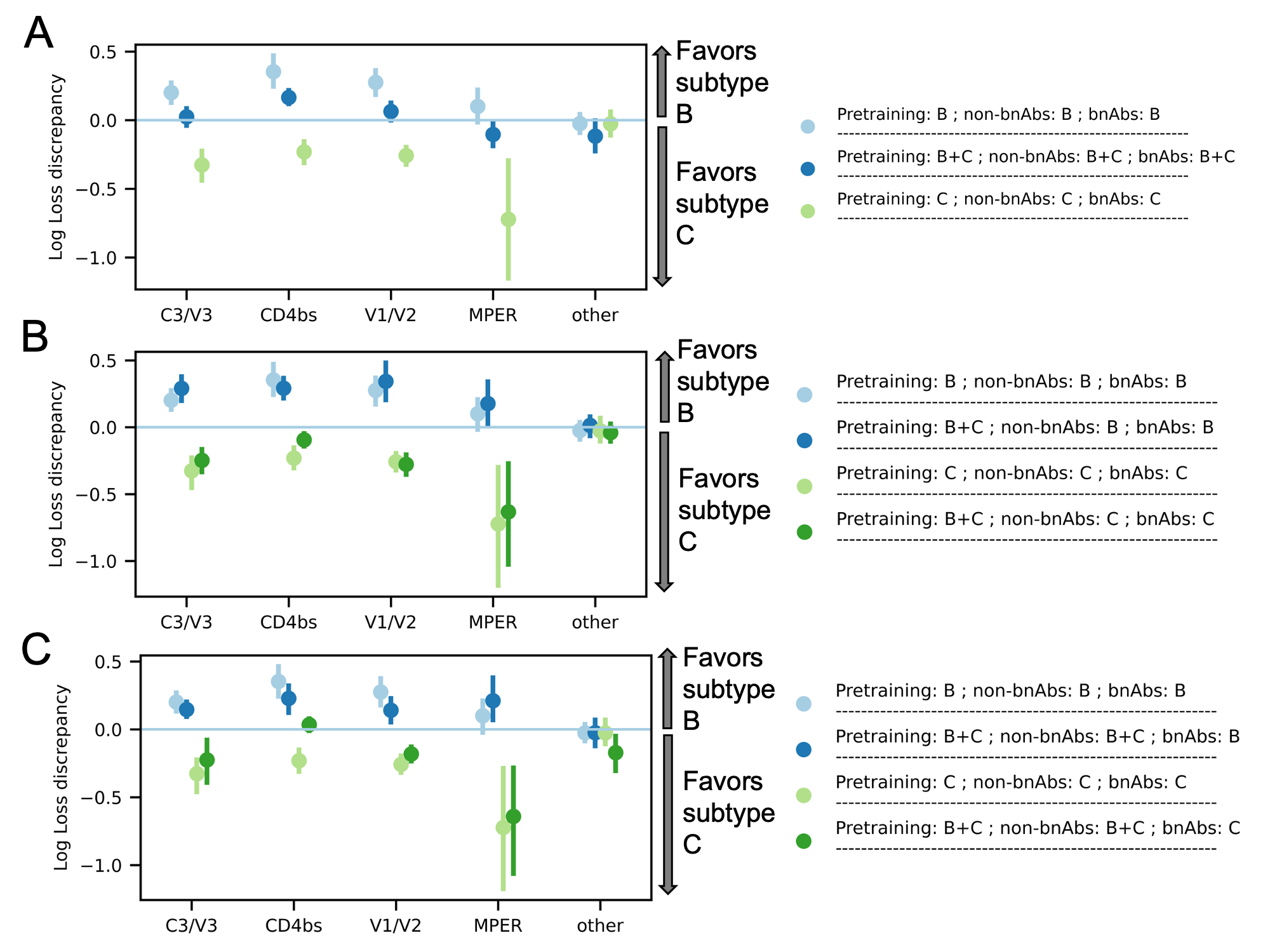

Supplement: S6 Fig — We named models according to subtype combinations contained in the pretraining data (shown as “Pretraining”), in data on non-bnAbs (shown as “non-bnAbs”), and in bnAb data (shown as “bnAbs”). Log Loss discrepancy means Log Loss on subtype C minus Log Loss on subtype B. (A) shows the bias introduced by only training on one subtype, and how that bias is eliminated by more subtype diversity. (B) shows that subtype representativeness in the pretraining data reduces subtype bias only to a small extent, if at all. (C) shows how subtype representativeness in non-bnAb data reduces subtype bias. Error bars represent the 95% confidence intervals computed using 1000 bootstrap samples. Models were trained on sequences paired with phenotypes defined using IC50. (TIFF) [file pcbi.1012618.s006.tiff]
